# Supplementary material for: NH2-MIL-53(Al) Polymer Monolithic Column for In-Tube Solid-Phase Microextraction Combined with UHPLC-MS/MS for Detection of Trace Sulfonamides in Food Samples
Source: Molecules. 2020 Feb 18;25(4):897. doi: 10.3390/molecules25040897 (PMC7070345; doi:10.3390/molecules25040897)
Supplement: Supplementary file 1 [file molecules-25-00897-s001.pdf]

NH_2-MIL-53(Al) polymer monolithic column for in-tube solid-phase microextraction combined with UHPLC-MS/MS for detection of trace sulfonamides in food samples

Qian-Chun Zhang*, Guang-ping Xia, Jun-yi Liang, Xiao-lan Zhang, Li Jiang, Yu-guo Zheng, Xing-yi Wang*

School of Biology and Chemistry, Key Laboratory of Chemical Synthesis and Environmental Pollution Control-Remediation Technology of Guizhou Province, Xingyi Normal University for Nationalities, Xingyi, 562400, P. R. China; xiaguangping@xynun.edu.cn (G.-P.X.); liangjunyi@xynun.edu.cn (J.-Y. L.); zhangxiaolan@xynun.edu.cn (X.-L. Z.); jiangli@xynun.edu.cn (L.J.); zhengyuguo@xynun.edu.cn (Y.-G. Z.)

*Correspondence: zhangqianchun@xynun.edu.cn (Q.-C. Z.); wangxingyi@xynun.edu.cn (X.-Y. W.) Tel: +86-589-3296359;

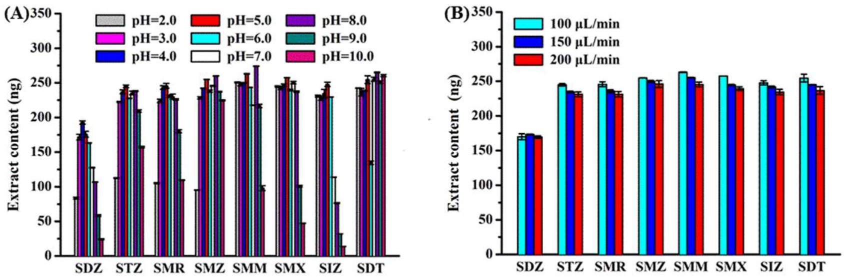

Fig. S1. Extraction conditions of the MOF-polymer monolithic column. Effect of the (A) pH of extraction solvent and (B) extraction flow rate.

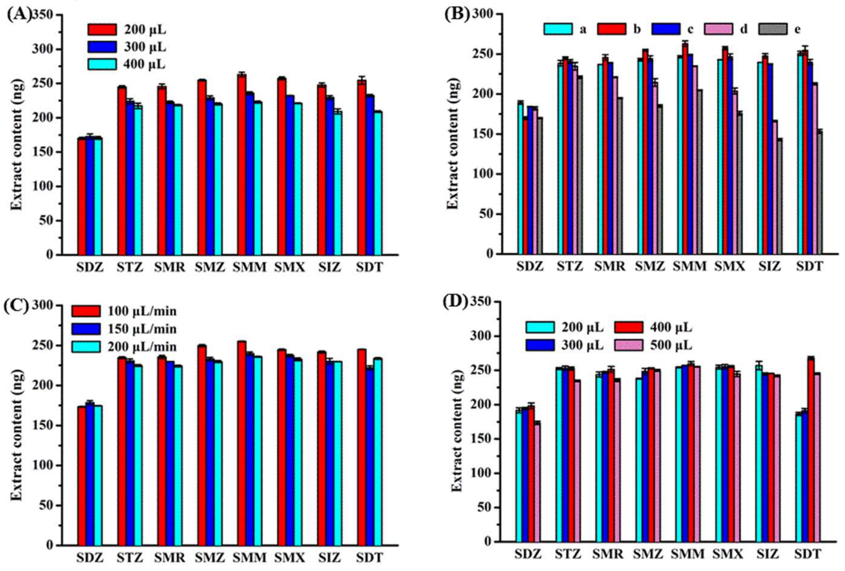

Fig. S2. Extraction conditions of the MOF-polymer monolithic column. Effect of the (A) purification volume, (B) desorption solvent, (C) desorption flow rate, and (D) desorption volume.

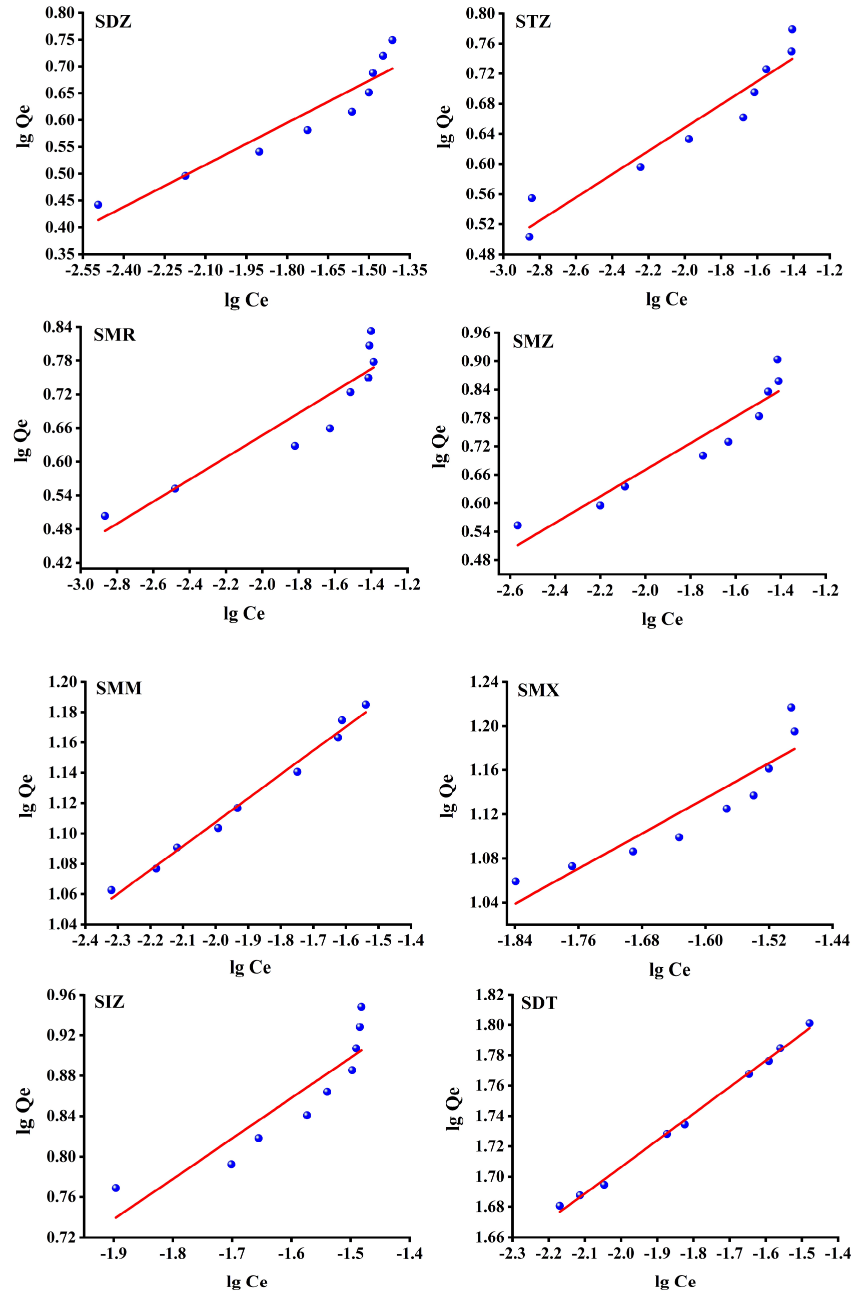

Fig. S3. Freundlich isotherm adsorption model curves of SAs.

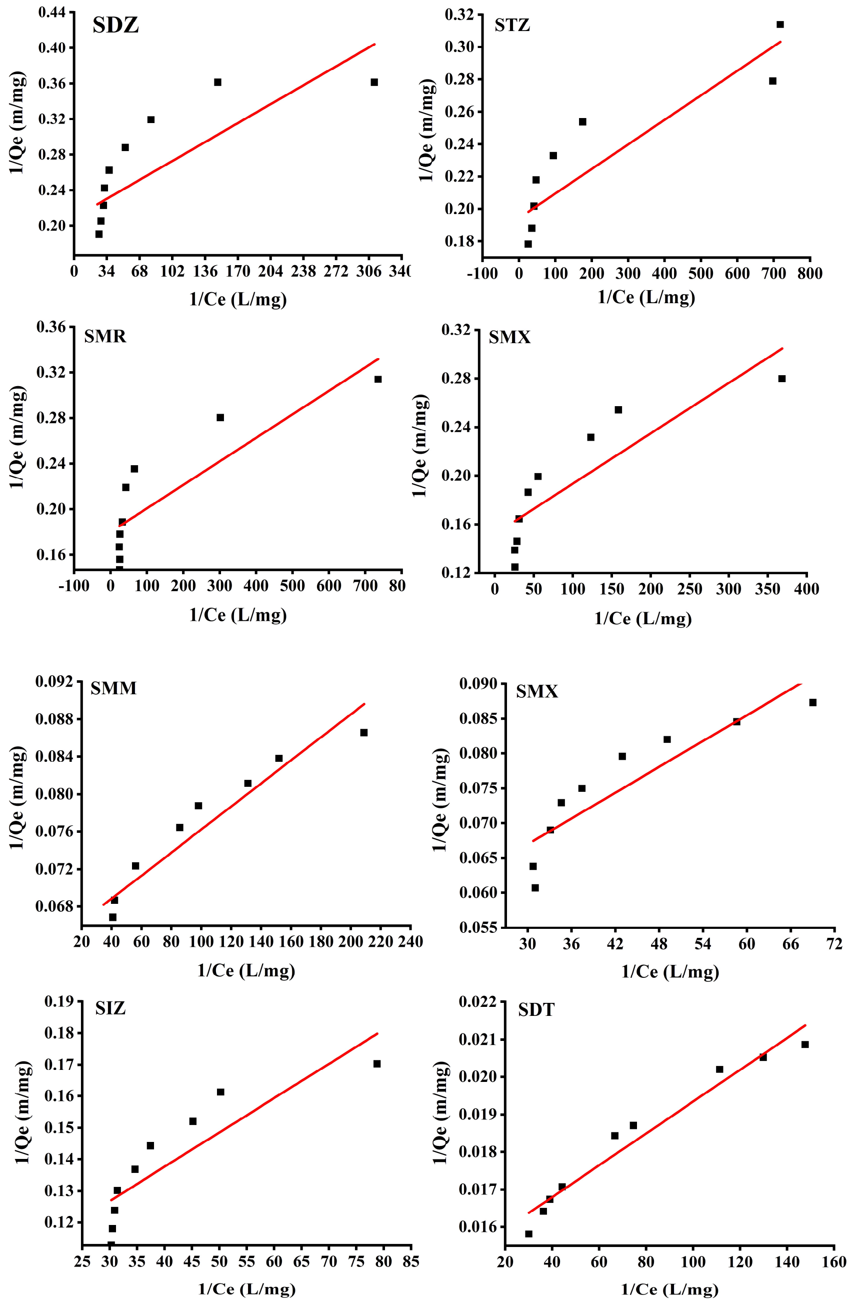

Fig. S4. Langmuir isotherm adsorption model curves of SAs.

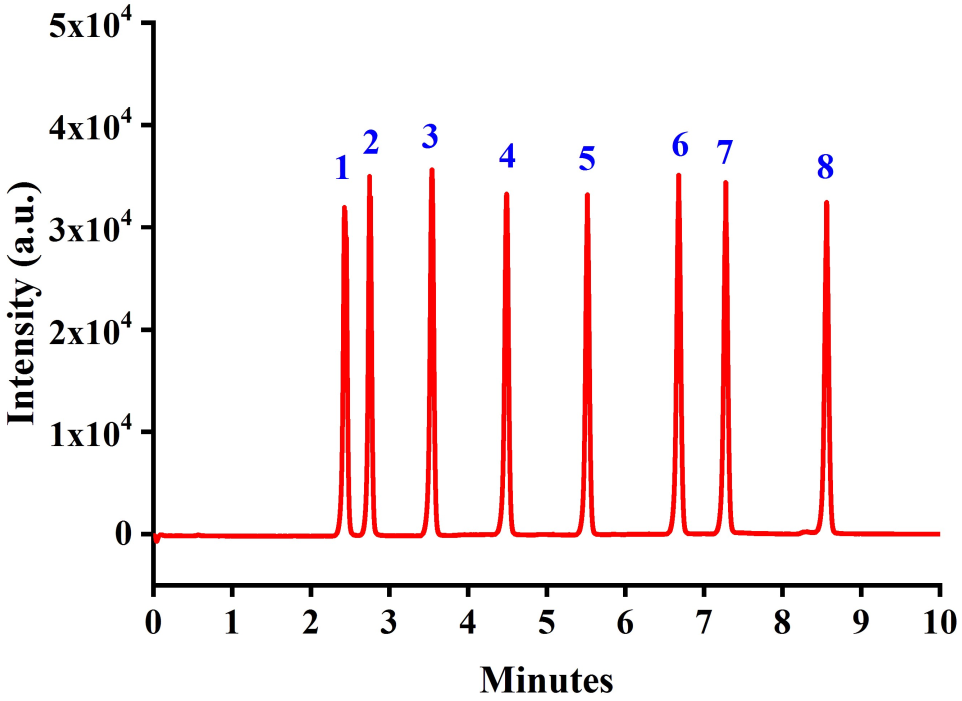

Fig. S5. Total ion chromatography of analytes with direction injection of the standard solution at 40.0 μg/L. 1: SDZ, 2: STZ, 3: SMI, 4: SMZ, 5: SMM, 6: SMX, 7: SIZ, 8: SDM

Table S1. Optimized MS/MS parameters for the parent and quantitative daughter ions (m/z) and collision energy (CE) of the eight SAs used in this study.

Analyte	Precursor $[m/z]^+$	Product $[m/z]^+$	Q1 Pre Bias （V）	CE（V）	Q3 Pre Bias （V）
SDZ	251.22	155.91	-12	-30	-27
		92.04	-12	-30	-15
STZ	256.15	156.05	-30	-25	-25
		92.04	-30	-24	-15
SMI	265.18	156.02	-16	-32	-26
		107.96	-25	-26	-16
SMZ	279.23	186.03	-15	-20	-16
		123.92	-22	-28	-25
SMM	281.12	155.97	-15	-26	-16
		126.10	-30	-26	-26
SMX	254.17	155.92	-29	-25	-14
		92.06	-26	-18	-16
SIZ	268.09	156.01	-28	-28	-27
		92.03	-24	-28	-13
SDM	311.24	156.03	-18	-20	-30
		108.01	-25	-22	-16
SMZ -D_4	282.41	186.22	-18	-16	-22
		160.13	-20	-16	-19
